# Inducible Resistance to Maize Streak Virus

**DOI:** 10.1371/journal.pone.0105932

**Published:** 2014-08-28

**Authors:** Dionne N. Shepherd, Benjamin Dugdale, Darren P. Martin, Arvind Varsani, Francisco M. Lakay, Marion E. Bezuidenhout, Adérito L. Monjane, Jennifer A. Thomson, James Dale, Edward P. Rybicki

**Affiliations:** 1 Department of Molecular and Cell Biology, University of Cape Town, Rondebosch, Cape Town, South Africa; 2 Institute of Infectious Disease and Molecular Medicine, University of Cape Town, Observatory, Cape Town, South Africa; 3 Centre for High-Performance Computing, Rosebank, Cape Town, South Africa; 4 Centre for Tropical Crops and Biocommodities, Queensland University of Technology (QUT), Brisbane, Queensland, Australia; 5 School of Biological Sciences and Biomolecular Interaction Centre, University of Canterbury, Christchurch, New Zealand; 6 Department of Plant Pathology and Emerging Pathogens Institute, University of Florida, Gainesville, Florida, United States of America; 7 Electron Microscope Unit, Division of Medical Biochemistry, Department of Clinical Laboratory Sciences, University of Cape Town, Observatory, Cape Town, South Africa; Naval Research Laboratory, United States of America

## Abstract

Maize streak virus (MSV), which causes maize streak disease (MSD), is the major viral pathogenic constraint on maize production in Africa. Type member of the *Mastrevirus* genus in the family *Geminiviridae*, MSV has a 2.7 kb, single-stranded circular DNA genome encoding a coat protein, movement protein, and the two replication-associated proteins Rep and RepA. While we have previously developed MSV-resistant transgenic maize lines constitutively expressing “dominant negative mutant” versions of the MSV Rep, the only transgenes we could use were those that caused no developmental defects during the regeneration of plants in tissue culture. A better transgene expression system would be an inducible one, where resistance-conferring transgenes are expressed only in MSV-infected cells. However, most known inducible transgene expression systems are hampered by background or “leaky” expression in the absence of the inducer. Here we describe an adaptation of the recently developed INPACT system to express MSV-derived resistance genes in cell culture. Split gene cassette constructs (SGCs) were developed containing three different transgenes in combination with three different promoter sequences. In each SGC, the transgene was split such that it would be translatable only in the presence of an infecting MSV’s replication associated protein. We used a quantitative real-time PCR assay to show that one of these SGCs (pSPLIT*re*p^III-Rb-^Ubi) inducibly inhibits MSV replication as efficiently as does a constitutively expressed transgene that has previously proven effective in protecting transgenic maize from MSV. In addition, in our cell-culture based assay pSPLIT*rep* ^III-Rb-^Ubi inhibited replication of diverse MSV strains, and even, albeit to a lesser extent, of a different mastrevirus species. The application of this new technology to MSV resistance in maize could allow a better, more acceptable product.

## Introduction

During the past decade a great deal of effort has been spent on the development of crops with transgenic resistance against a number of different economically-important pathogenic single-stranded DNA (ssDNA) viruses in the family *Geminiviridae* [1]–[4]. Whereas much of the early work focused on pathogen-derived resistance approaches involving the expression of virus-derived genes in plants (see [3], [4] for reviews), more recent innovations have seen the application of interfering peptides such as recombinant peptide aptamers [5], [6] and zinc finger proteins [7]. All of these approaches have relied on constitutive expression of recombinant proteins, which can have several drawbacks: (1) constitutive expression of resistance genes is redundant when no viral infection occurs and will add unnecessarily to the metabolic load of uninfected transgenic plants; (2) constitutively expressed genes are more likely to be targeted for transgene silencing than inducible genes; (3) constitutive expression limits the types of transgene that can be used to those whose expression is not detrimental or toxic to plant cells. This last point is particularly pertinent since plants are usually transformed as cells or immature embryos in tissue culture and the expression of toxic gene products can therefore inhibit the regeneration of whole plants.

One way to overcome these problems would be to either delay expression of transgenes until plants have regenerated fully, or, in the case of virus resistance, to make transgene expression inducible only upon viral infection. This has been attempted previously for the geminivirus-induced expression of the cytotoxic ribosome inactivating protein dianthin [8] and the ribonuclease barnase from *Bacillus amyloliquefaciens* [4], [9], [10]. Both of these proteins are lethal when expressed in plant cells, and therefore can be used to mimic innate hypersensitivity responses to virus infection. However, because of their toxicity such genes need to be “switched off” in the absence of virus infections. In the case of barnase, this was achieved by co-expressing the extracellular barnase with its intracellular inhibitor barstar, which then bind to each other with high affinity [11], [12]. If produced at similar levels, barstar inhibits the expression of barnase, resulting in no RNase production. By placing barnase under control of a viral promoter that is activated upon viral infection, and barstar under a viral promoter that is repressed upon viral infection, Zhang et al. [10] surmised that over-expression of barnase relative to barstar would kill virus infected cells, thus preventing further virus spread. The strategy attempted by Hong et al. [8] to express dianthin was a similar one: the gene was placed under control of a viral promoter that is activated by the begomoviral transcriptional activator protein (TrAP).

Despite being promising options for inducible transgene expression, these strategies have certain drawbacks. With both dianthin and barnase, “leaky” or low-level basal expression from the viral promoter occurs in the absence of the viral TrAP ([8], [10]. In addition, Hussain et al. [13] have shown with Tomato leaf curl New Delhi virus (ToLCNDV) that the hypersensitive response naturally triggered in *Nicotiana tabacum* and *Lycopersicon esculentum* plants by the ToLCNDV nuclear shuttle protein (NSP) [14] is suppressed by TrAP. If other geminiviruses encode similar anti-hypersensitive response factors it may undermine cell death-inducing resistance mechanisms.

**Figure 1:**
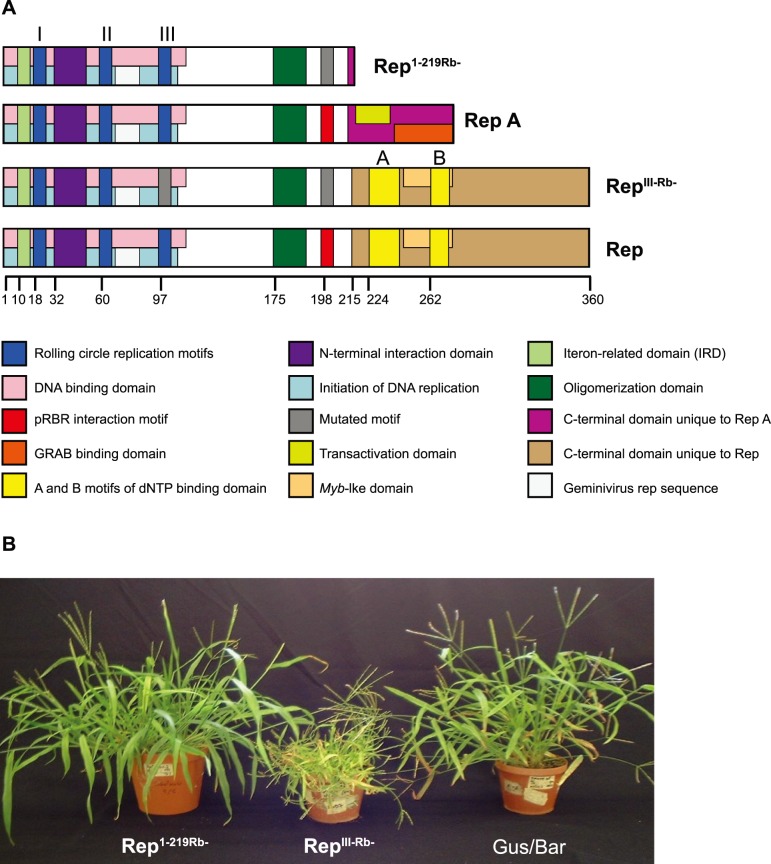
Products of mutated and truncated MSV *rep* genes used in the split gene cassettes, compared with the wild type. A) Known sequence motifs and functional domains present in each gene product are highlighted. Amino acid numbering is relative to the N-terminal methionine. Adapted from Shepherd et al. [17]. B) Three representative *Digitaria sanguinalis* lines constitutively expressing p*rep* ^1–219Rb-^ (left), p*rep* ^III-Rb-^ (middle) or Gus and Bar (from pAHC25 [39]; right), illustrate the phenotypic effects of the transgenes. Photo from Shepherd et al. [17].

Maize streak disease (MSD), caused by the geminivirus species *Maize streak virus* (genus *Mastrevirus*), results in substantial maize yield reductions throughout sub-Saharan Africa and in some years can cause regional maize crop failures [15]. Throughout the African continent the development of MSD-resistant maize varieties is therefore a prime objective for both conventional maize breeders and biotechnologists. While we have had success in using a constitutively expressed “dominant negative” mutated and truncated replication associated protein (*rep*) transgene to provide resistance to MSV in maize (*rep* ^1-219Rb-^ [16]), subsequent research has indicated that far greater degrees of MSV resistance are potentially achievable. In our initial screen of a range of *rep*-derived transgenes, first in a transient expression assay using maize suspension cells, and second in the model plant *Digitaria sanguinalis* [17], we found that a full-length *rep* gene containing mutations in the rolling circle replication (RCR) motif III and retinoblastoma related protein binding domain, pRBR (*rep* ^III-Rb-^; Fig. 1A) provided much better resistance against MSV than the truncated version of this gene (all challenged plants were immune); however, we did not progress with this construct because its constitutive expression also led to stunting and infertility in transgenic plants (Fig. 1B).

**Figure 2:**
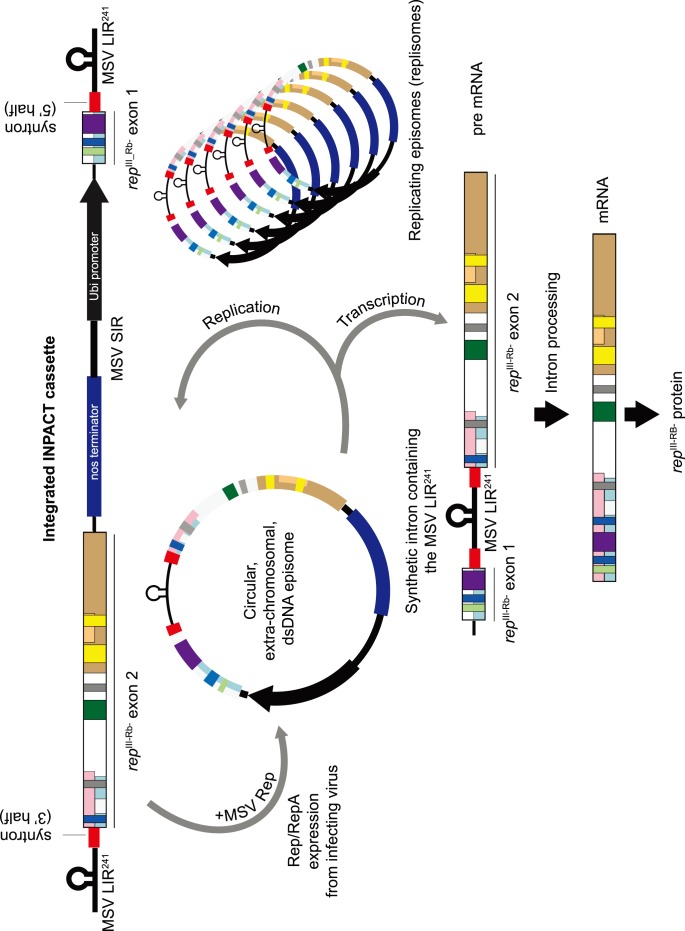
Schematic representation of the INPACT system. MSV-inducible expression from a “split gene cassette” using pSPLIT*rep* ^III-Rb-^Ubi is used as an example. NosT = nopaline synthase terminator; UbiP = maize ubiquitin promoter. MSVLIR^241^– =  truncated MSV long intergenic region.

For MSV-inducible expression of the *rep*-derived transgenes, we developed constructs called “split gene cassettes” (SGCs), based on a novel protein production platform known as INPACT (In Plant Activation [18], [19]). These cassettes are arranged such that the gene of interest is split into two exons and the transgene cannot be expressed in the absence of the MSV Rep. Each SGC (Fig. 2) is flanked by two virus-derived long intergenic regions (LIRs), which contain the virion-sense strand origin of replication and Rep binding and nicking sites [20]–[24], which are in turn embedded within a small synthetic intron termed a syntron [18], [19]. The cassettes also include the mastreviral short intergenic region (SIR) which contains the origin of complementary-strand synthesis [25]–[27]. Upon viral infection the integrated cassette serves as a template for RCR, allowing replicative release [24] and amplification of circular ssDNA forms. Conversion to the dsDNA intermediate form occurs via the SIR using host cell machinery, after which the transgene is transcribed. Removal of the LIR-containing syntron during mRNA processing results in the reconstitution of a translatable in-frame transcript of the gene of interest (Fig. 2).

Replicative release of the integrated construct from the plant genome relies on the specific DNA nicking and joining activities of the MSV Rep, which recognises and binds to sequence-specific repeats known as iterons in the LIR. Because MSD is caused by only one maize-adapted strain, MSV-A [28], Rep-iteron specificity should not be a drawback in terms of obtaining broad resistance to MSD, but will provide an advantage in that functional proteins should only be produced in the presence of mastrevirus Reps that are sufficiently similar to that of MSV-A. This may overcome the problems associated with leaky inducible promoters reported with other systems.

Here we use a cell-culture based assay to demonstrate that, in addition to this inducible transgene expression system being capable of providing particularly high degrees of resistance against MSV-A, it could also potentially provide transgenic maize with broad, albeit less potent, resistance both against diverse grass-adapted MSV strains and other African mastrevirus species such as *Panicum streak virus* (PanSV).

## Materials and Methods

### Construct Design

#### Truncation of the MSV Long Intergenic Region and Assaying for Cryptic Splice Sites

The first step in designing the SGCs was to truncate the 3′ terminus of the MSV LIR by 70 bp to remove the virion (V) sense promoter region, thus avoiding the possibility of trans-activation of the V-sense promoter and unwanted transcript expression. Primers were designed to amplify a 5′-terminal 241-bp sequence stretch from the LIR of MSV-A4 [ZA-Kom-1989] ([29]; GenBank accession no. AF003952); hereafter referred to as MSV-Kom. This region contains the minimum LIR sequence required for RCR, as determined by Willment et al. [30], and consists of a stem-loop structure and nicking site essential for the initiation of RCR by Rep [22], [31], as well as iterons for Rep-binding [32]–[36]. *Pac*I and *Swa*I restriction enzyme (RE) sites were incorporated at the 5′ terminus of the forward and reverse primers respectively to flank the amplified product for future cloning (Table 1). The PCR product was ligated with pGEMT-Easy (Promega) and sequenced at Macrogen Inc., Korea.

**Table 1:** Primer sequences.

Primer name	1Sequence (5′-3′)
*Primers for SGC cloning*	
LIR^241^ *Pac*I (F)	TTAATTAA GCCGACGACGGAGGTTGAGG
LIR^241^ *Swa*I (R)	ATTTAAAT CATACAAAGCAGAACCAGGC
GUSex1*Bam*HI (F)	GGATCC ATGGTACGTCCTGTAGAAACCCCAACCCG
GUSex1 (R)	GAGTTTCATCGTACGGTACTTGAG
GUSex2 (F)	GTGCGCCGTAGTTTCCTTTAG
GUSex2*Spe*I (R)	ACTAGT TTATTGGAGATCCTCATTGTTTGC
Ubi*Asc*I (F)	GGCGCGCC AAGCTTGCATGCCTGCAGTGCAG
Ubi*Bam*HI (R)	GGATCC TCTAGAGTCGACCTG
UbiΔI*Bam*HI (R)	GGATCC AGAGGGTGTGGAGGGGGTGTCTATTTATTACG
*Real-time PCR Primers*	
MSV-Kom Rep (F)	TTGGCTGTCAGAGGGATTTC
MSV-Kom Rep (R)	CCCTGGAGTCATTTCCTTCA
MSV-Kom CP (F)	TAAGCGGGTGCCTAAGAAGA
MSV-Kom CP (R)	TGCTGGAGTGTCTGGATTTG
MSV-VW CP (F)	GGGAGATGATTCGAACTGGA
MSV-VW CP (R)	TGCTGGAGTGTCTGGATCTG
MSV-Set CP (F)	AGTTGTGTCATCGCTTCGTG
MSV-Set CP (R)	TGGTGTATCCGAGCCTATCC
PanSV-Kar CP (F)	CCACACCAACGAGACTCTGA
PanSV-Kar CP (R)	CAACCACATGACACCCACTC
Maize18S (F)	CAGGGATCAGCGGTGTTACT
Maize 18S (R)	GGTAAGTTTCCCCGTGTTGA

1 Underlined letters highlight engineered restriction enzyme (RE) sites (names of the introduced RE sites are incorporated in the primer names).

**Figure 3:**
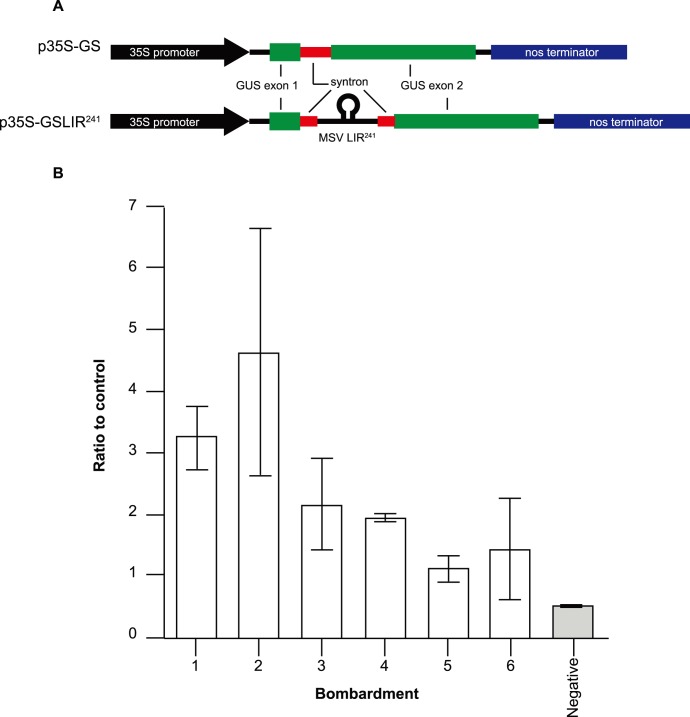
Gus assays to test for cryptic splice sites in the MSV long intergenic region. A) Gus expression cassettes used in the assays. B) Expression of Gus from p35S-GSLIR^241^ (test construct) as a ratio to p35S-GS (positive control construct), four days after bombardment. Each bar is an average of three replicates; error bars represent 95% confidence intervals. Negative = negative control (protein extract from a non-bombarded Black Mexican sweet sample).

The second step was to test the MSV LIR^241^ sequence for potential intron splice sites, which may cause problems during processing of the functional mRNA when the construct is replicationally released by the viral Rep. To do this, PCR-amplified LIR^241^ was embedded within a synthetic intron (syntron) developed at Queensland University of Technology [18], [19]. The LIR-containing syntron was in turn embedded within the GUS reporter gene coding region of a pUC19-based expression cassette (CaMV35S-promoter>GUS>CaMV35S-terminator), thus splitting the coding region into two exons and creating vector p35S-GSLIR^241^ (Fig. 3A). After bombardment of p35S-GSLIR^241^ into Black Mexican sweet (BMS) maize suspension cells using a Bio-Rad PDS-1000/He particle gun (following the methodology of Shepherd et al. [37]), GUS expression was compared with a control construct containing the syntron with no embedded LIR (p35S-GS; Fig. 3A). This was to determine if p35S-GSLIR^241^ expressed the same or similar level of GUS as did the p35S-GS control vector. Lower expression could mean there is a cryptic 3′-terminal splice site in the LIR that interferes with syntron splicing and subsequent GUS translation, while similar expression would indicate no such problem.

Crude protein was extracted from bombarded BMS cells using the GUS extraction buffer from the Marker Gene Technologies (MGT) β-Glucuronidase (GUS) Reporter Gene Activity Detection Kit according to the instruction manual protocol: (http://search.cosmobio.co.jp /cosmo_search_p/search_gate2/docs/MGT_/M0877.20080313.pdf).

Protein in these crude extracts was quantified using the BioRad Protein Assay kit (http://labs.fhcrc.org/fero/Protocols/BioRad_Bradford.pdf) and each sample was diluted to a concentration of 2 mg/ml.

GUS activity was measured using the above-mentioned MGT reporter gene kit according to the kit instructions. The fluorogenic substrate, methylumbelliferyl b-D-glucuronide (4-MUG), was used at a final molarity of 0.04 mM (40 µl of 0.1 mM 4-MUG in 100 µl total volume); while the final concentration of each protein extract (six samples bombarded with p35S-GS; six samples bombarded with p35S-GSLIR^241^, and one non-bombarded BMS control sample) was 0.2 mg/ml (10 µl of 2 mg/ml extract in a total volume of 100 µl). Fluorescence was measured using a Cary Eclipse Fluorescence Spectrophotometer (Agilent) with emission and excitation filters set at 455 nm and 365 nm respectively. For each test sample, three replicates and two blanks (GUS extraction buffer in place of protein extract) were assayed. “Test” fluorescence was subtracted from “blank” fluorescence for all samples, and then a ratio was calculated of p35S-GSLIR^241^ to p35S-GS. Ratios below 1 would indicate interference with GUS expression possibly due to the presence of cryptic splice sites in the LIR^241^. A Mann Whitney test (GraphPad Prism) was used to determine any significant differences in GUS expression between the test and control constructs.

#### Construction of Split Gene Cassette Constructs

**Figure 4:**
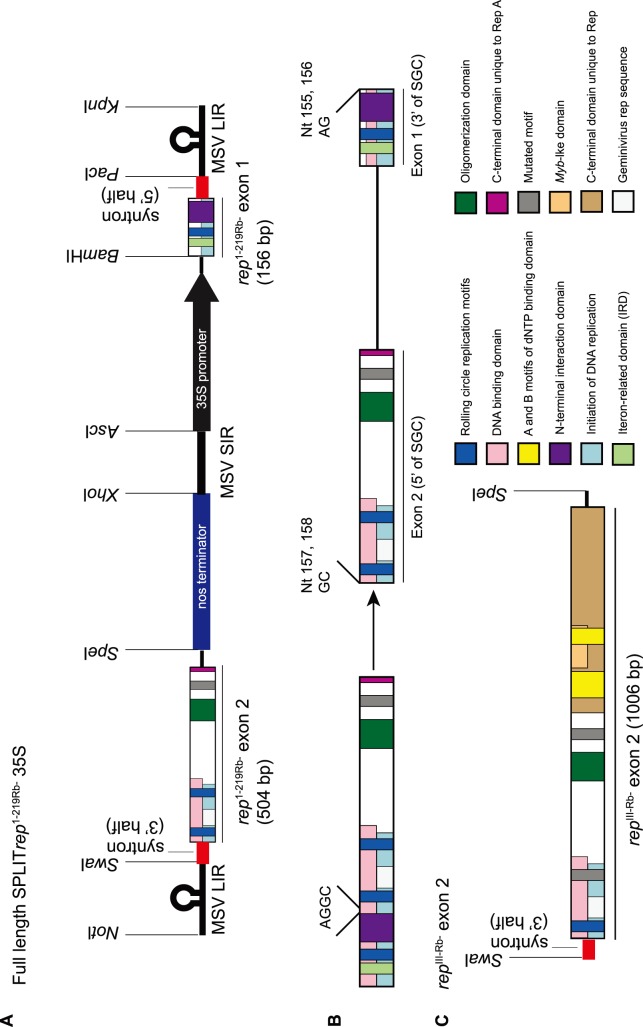
Schematic diagram of synthesised constructs, with restriction enzyme sites incorporated for subsequent cloning. A) pSPLIT*rep* ^1-219Rb-^35S containing “modules” that could be removed and replaced with other sequences by restriction digest. B) Illustration showing how the *rep* ^1-219Rb-^ transgene was split at the first AGGC (nucleotides 155, 156, 157 and 158 with respect to the start codon). The exon 2, cloned at the 5′ terminus of the split gene cassette in A) therefore began with GC, and the exon 1, cloned at the 3′ terminus, ended in AG. C) The synthesised *rep* ^III-Rb-^ (see Fig. 1A for the full-length gene product) exon 2, preceded by the 3′-terminal half of the syntron, flanked by *Swa*I and *Spe*I RE sites. The 3′-terminal syntron/*rep* ^1-219Rb-^ exon 2 in pSPLIT*rep* ^1-219Rb-^35S was replaced by the 3′-terminal syntron/*rep* ^III-Rb-^ exon 2 to create pSPLIT*rep* ^III-Rb-^35S. Exon 1 remained the same for both constructs since they share the same 5′-terminal 156 bp. Similarly, other modules were exchanged to create further constructs, such as the CaMV 35S promoter for the maize ubiquitin promoter etc (see text for details).

A full-length SGC (pSPLIT*rep* ^1-219Rb-^35S; Fig. 4A and Fig. S1) was synthesised at Epoch Life Science Inc (USA) who provided it cloned in the *Sma*I site of pBluescript II SK (pSK; Stratagene, USA). As part of the construct design, *Not*I and *Kpn*I RE sites flanked the SGC to enable the removal of the entire cassette from pSK (Fig. 4A). The synthesised SGC was designed such that each feature or “module” (e.g. the promoter, terminator, exon 1 or exon 2 sequences) can be removed and replaced with other sequences by restriction digest. However, for downstream cloning purposes some RE sites in the pSK multiple cloning site had to be removed (e.g. the *Bam*HI site, which needed to be unique to the SGC for subcloning of both the promoter and exon 1 sequences; see Fig. 4A). This was achieved by removing the *Sma*I-cloned SPLIT*rep* ^1-219Rb-^35S cassette with *Not*I/*Kpn*I and re-cloning it into the *Not*I/*Kpn*I sites of pSK, in the process removing the portion of the multiple cloning site that was sandwiched between the *Kpn*I and *Not*I sites. This was then used as the backbone for the cloning of a further eight constructs.

To generate split exon 1 and exon 2 sequences, the *rep* ^1-219Rb-^ coding region of pSPLIT*rep* ^1-219Rb-^35S was designed such that it was split at the first AGGC to create exon 1 (ending in AG at position 155/156, with position 1 being the start codon), and exon 2 (beginning with GC at position 157/158) (See Fig. 4B).

For the cloning of the full-length *rep* ^III-Rb-^ SGC, the exon 2 fused to the 3′-terminal half of the syntron was also synthesised, with *Swa*I and *Spe*I RE sites flanking the fragment (Fig. 4B). Thus, the *rep* ^1-219Rb-^ exon 2 in pSPLIT*rep* ^1-219Rb-^35S could be replaced with that of *rep* ^III-Rb-^ using the *Swa*I/*Spe*I RE sites. Exon 1 remained the same in both constructs, since both *rep* ^1-219Rb-^ and *rep* ^III-Rb-^ share the same 5′-terminal 295 bp.

For the GUS constructs, a previously made GUS-based SGC (pINPACT-GUS; [18]) was used as template for PCR amplification (see Table 1 for primer sequences) of the 3′-terminal syntron/GUS exon 2 and the GUS exon1/5′-terminal syntron. The design of pINPACT-GUS is essentially the same as for the MSV-based SGC shown in Fig. 4A, except that truncated Tobacco yellow dwarf virus (TYDV) LIRs flank the construct, GUS exon 1 and 2 are in place of MSV *rep*-derived exon 1 and 2, and some of the RE sites flanking each “module” differ. Also, the GUS coding region is split at the first AGGT to create exon 1 (ending in AG at position 231/232, relative to the GUS start codon) and exon 2 (starting with GC at position 233/234).

For amplification of the 3′-terminal syntron/GUS exon 2 from pINPACT-GUS, the forward primer, GUSex2 (F), was designed to anneal to the last 3′-terminal 21 nucleotides of the TYDV truncated LIR, while the reverse primer, GUSex2*Spe*I (R), incorporated an *Spe*I RE site at the 3′ terminus of the GUS exon 2. Since the 3′-terminal half of the syntron starts with a *Swa*I site, the amplified 3′-terminal syntron/GUS exon 2 could be cloned into the *Swa*1/*Spe*I sites of pSPLIT*rep* ^1-219Rb-^35S (See Fig. 4A).

For the GUS exon1/5′-terminal syntron amplification, a *Bam*HI site was incorporated at the 5′ terminus of the forward primer, GUSex1*Bam*HI (F), while the reverse primer, GUSex1 (R) was designed to anneal to the 5′-terminal 24 bp of the TYDV truncated LIR of pINPACT-GUS. The 5′-terminal half of the syntron ends with a *Pac*I site; thus the amplified GUS exon1/5′-terminal syntron could be cloned into the *Bam*HI/*Pac*I sites of pSPLIT*rep* ^1-219Rb-^35S.

Since the synthesised pSPLIT*rep* ^1-219Rb-^35S was used as the backbone for the cloning of *rep* ^III-Rb-^ and GUS exons 1 and 2, all three SGCs contained the CaMV35S promoter. However, we wanted to test two additional promoter combinations: the maize ubiquitin promoter (ubi-1) complex, which includes the first intron of the maize ubiquitin-1 gene as well as an untranslated exon for enhanced expression in maize [38] and the maize ubi-1 promoter without the exon and intron. Because splicing of the syntron needs to occur in order to fuse exons 1 and 2 of the transgenes, we were uncertain whether the presence of a second intron (within the promoter region) would interfere with this, hence testing an “intronless” ubi-1 promoter.

Both the ubi-1 promoter complex (simply called Ubi) and the ubi-1 promoter without the exon/intron (called UbiΔI) were PCR amplified from pAHC17 [39] with the addition of flanking *Asc*I and *Bam*HI RE sites (See Fig. 4A and Table 1). The same forward primer (Ubi*Asc*I [F]), but different reverse primers (Ubi*Bam*HI [R] and UbiΔI*Bam*HI [R]) were used for amplification of Ubi and UbiΔI promoters respectively (see Table 1 for primer sequences).

The CaMV35S promoter in *rep* ^1-219Rb–^, *rep* ^III-Rb–^ and GUS-based SGCs was then replaced by (a) Ubi and (b) UbiΔI, resulting in a total of nine SGCs. These were called (1) pSPLIT*rep* ^1-219Rb-^35S; (2) pSPLIT*rep* ^1-219Rb-^Ubi; (3) pSPLIT*rep* ^1-219Rb-^UbiΔI; (4) pSPLIT*rep* ^III-Rb-^35S; (5) pSPLIT*rep* ^III-Rb-^Ubi; (6) pSPLIT*rep* ^III-Rb-^UbiΔI; (7) pSPLITGUS35S; (8) pSPLITGUSUbi; (9) pSPLITGUSUbiΔI. While all constructs were tested initially in a qualitative PCR assay, only Ubi- and UbiΔI-containing SGCs were assayed by quantitative PCR.

### Inoculation of Maize Suspension Cells

To rapidly assay the effectiveness of the various SGCs in inhibiting MSV replication, maize suspension cells were bombarded with each SGC and a partial dimer (1.1 mer) of the MSV-Kom genome (pKom602; [40]). MSV-Kom, the isolate from which the *rep*, LIR and SIR sequences in the SGCs were derived, belongs to an MSV-A subtype known as MSV-A_4_, which is the most prevalent subtype found in South Africa [41].

BMS suspension-cultured cells were subcultured at a 1∶3 dilution three days prior to bombardment. Twenty-four hours before bombardment, 1.0 mL packed volume of actively dividing cells was plated onto solid media.

Different combinations of plasmid DNA (described below) were precipitated onto 1 µm gold particles (50 µl of 60 mg/ml gold suspended in 50% glycerol) according to the protocol of Dunder et al. [42], and these were delivered into the plated BMS cells using the PDS-1000/He Biolistic particle bombardment delivery system (Bio-Rad) using the method of Shepherd et al. [37]. After bombardment, plates were incubated at 25°C in the dark for four days, after which total DNA was extracted from the BMS cells as described [37].

Initially, each SGC was co-bombarded with pKom602 at a 1∶1 weight ratio (as in Owor et al., [43]); i.e. 2 µg of each plasmid per 50 µl gold precipitation. Subsequently, only the Ubi- and UbiΔI-containing SGCs were assayed, this time at SGC:pKom602 weight ratios of 1∶1 and 5∶1 (2 µg of each plasmid for a 1∶1 ratio; 2 µg of pKom602 and 10 µg of SGC for a 1∶5 ratio). pSPLIT*rep* ^III-Rb-^Ubi was then tested against cloned MSV isolates belonging to the B and C strains of MSV: MSV-B1 [ZA-VW-Triticum-1993] and MSV-C [ZA-Mt Edg-Setaria-1988] (Genbank accession numbers AF239960 and AF007881 respectively), hereafter referred to as MSV-VW and MSV-Set, respectively. In addition, the effectiveness of the SGC was tested against a different species of Mastrevirus, PanSV-A [ZA-Kar-1989] (GenBank accession number L39638), hereafter referred to as PanSV-Kar [40], [44], [45].

In each bombardment experiment, six plates were bombarded with the infectious mastrevirus clone (MSV-Kom, MSV-Set, MSV-VW or PanSV-Kar) alone, six plates were bombarded with each mastrevirus + pSK (empty vector), and nine plates were bombarded with each mastrevirus + SGC. A non-bombarded BMS plate was always included as a negative control. Each of these experiments was repeated at least twice.

### Quantitative Realtime PCR

Quantitative real-time PCR (qPCR), using a Rotor gene RG-3000A device (Qiagen, USA) and SYBR Green I (KAPA SYBR FAST qPCR kit, KAPA Biosystems, South Africa), was performed to determine viral titres in bombarded samples four days post-bombardment. Depending on the level of viral DNA in each sample (initially estimated using replicative-form specific conventional PCR as described by Owor et al. [43]), either 10 ng or 50 ng total DNA was used as template. The realtime PCR was carried out essentially as described [43], except that different primer pairs were used for detection of different viral genotypes (see Table 1). Separate standards were made for each viral genotype, using cloned MSV-Kom, MSV-Set, MSV-VW or PanSV-Kar. In each case, viral plasmid concentrations were 1000, 100, 10, 1, 0.1 and 0.01 pg/ul. As in Owor et al. [43], Maize18S (F) and Maize18S (R) primers were used to amplify a 173 bp product from the *Zea mays* 18S small subunit rRNA gene for normalization of data from different runs. For the 18S standard curve, BMS genomic DNA was gel quantified and diluted to 100, 50, 25, 10, 5 and 1 ng/ul.

Data were analysed using the computer program Rotor-Gene, version 6. Data were used only if amplification efficiencies calculated by the program were above 80% and Pearson’s correlation coefficient, *r* ^2^, of the standard curves was 0.99 or above. Viral plasmid and 18S standard curves were included in each run, rather than importing a previously performed standard curve.

### Statistical Analysis

Real-time PCR data were imported from Rotor-Gene version 6 into Microsoft Excel 2007 for calculation of the virus titres present in each sample. Further statistical analyses (Mann–Whitney tests) were carried out using GraphPad, version 5. Because multiple datasets were compared, a step-down multiple testing correction step was used when calculating P values.

## Results and Discussion

### Assay for cryptic splice sites within the MSV truncated LIR^241^

The 5′-terminal 241 nucleotides of the MSV-Kom LIR, previously determined to contain all the viral genomic *cis*-acting elements necessary for first strand synthesis [30] were assayed for any potential splice sites that could interfere with splicing of the syntron in which the LIR is embedded. As can be seen in Fig. 3B, GUS expression directed by p35SGSLIR^241^ (Fig. 3A), four days post-bombardment, was not reduced in six independent experiments in comparison to the positive control construct p35SGS. All GUS expression ratios in comparison to the positive control were above 1, although this increase in expression was not significant (p = 0.1143; Mann Whitney test). This indicated that embedding the LIR^241^ within the syntron of the SGCs will not have an appreciable effect on the splicing of the syntron that is required for fusion of the exon 1 and exon 2 sequences prior to their expression.

### Viral replication inhibition by split gene cassette constructs

**Figure 5:**
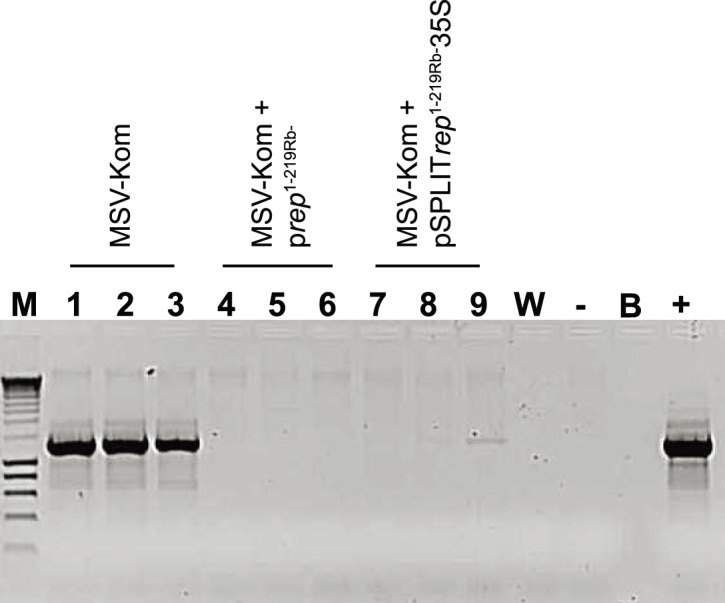
Replicative-form specific end-point PCR assay to test the effectiveness of the synthesised split gene cassette, pSPLIT*rep* ^1-219Rb-^35S, in interfering with MSV replication. Black Mexican sweet (BMS) cells were bombarded with an infectious clone of MSV-Kom (pKom602) alone (lanes 1–3); pKom602 and pSPLIT*rep* ^1-219Rb-^35S (lanes 7–9), as well as pKom602 and p*rep* ^1-219Rb-^ (constitutively expressed from the maize ubiquitin promoter [16], [17]) for comparative purposes (lanes 4–6). W = water control, − = non-bombarded BMS control, + =  positive control (pKom602 plasmid DNA). B = blank. The PCR was performed on total DNA extracted from BMS cells four days post-bombardment.

The basic design of the SGCs is illustrated in Figs. 2 and 4 and is described fully by Dugdale et al. [18], [19]. Since pSPLIT*rep* ^1-219Rb-^35S was the first to be synthesised and was the template upon which the rest of the SGCs were based, we first tested its capacity to inhibit MSV-Kom replication, using a replicative-form specific semi-quantitative end-point PCR assay [17]. Co-bombardment of pKom602 (a cloned 1.1-mer of the MSV-Kom genome; [40]) with pSPLIT*rep* ^1-219Rb-^35S was compared with co-bombardment of pKom602 with a construct constitutively expressing Rep^1-219Rb-^, which has been shown to inhibit viral replication in transgenic maize plants [16]. As can be seen in Fig. 5, viral titres were reduced in maize suspension cultures bombarded with each of p*rep* ^1-219Rb-^ and pSPLIT*rep* ^1-219Rb-^35S. Confident that the SGC system was working as expected and that expression of *rep* ^1-219Rb-^ from the SGC was occurring at a high enough level to inhibit viral replication (indicating that virus-mediated replicative release, circularisation, transcription and syntron splicing had all likely occurred effectively), we went ahead and constructed the remaining eight SGCs.

**Figure 6:**
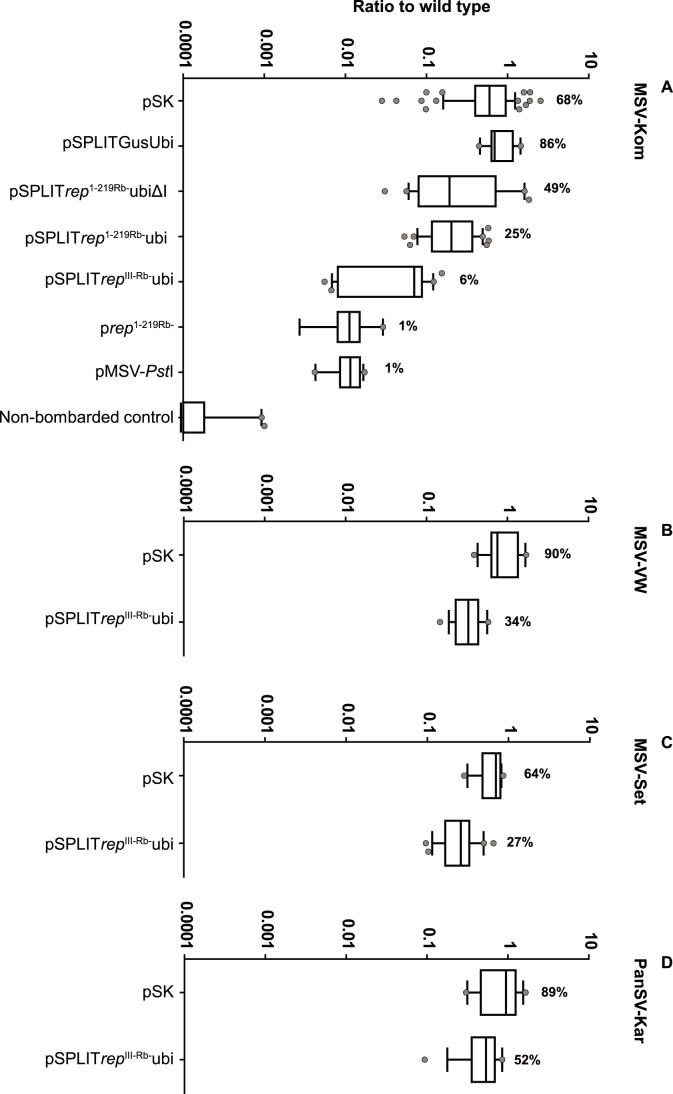
Vertical box-and-whisker plots summarising real-time PCR data on all constructs bombarded at a 1∶1 weight ratio with infectious clones of diverse MSV strains and another mastrevirus species. A) MSV-Kom. The plots show the sample minimum and maximum, the lower quartile (25th percentile; bottom of box), the median (50th percentile; horizontal line in box) and the upper quartile (75th percentile; top of box). The whiskers indicate the 10^th^ –90^th^ percentile: any data points outside of this are shown as dots. The y-axis (on a log_10_ scale) shows the ratio of MSV-Kom + construct to MSV-Kom alone (wild type). A value of <1 indicates a reduction in virus replication. Numbers above each plot are percent replication means compared with wild type. The number of replicates used to construct the plots (i.e. the number of bombarded samples) were as follows: pSK, 75; pSPLITGusUbi, 11; pSPLIT*rep* ^1-219Rb-^UbiΔI, 23; pSPLIT*rep* ^1-219Rb-^Ubi, 39; pSPLIT*rep* ^III-Rb-^Ubi, 21; p*rep* ^1-219Rb-^, 9; pMSV-PstI, 14; Non-bombarded control, 21. Plots in B-D) were constructed as described for A), but this time either pSPLIT*rep* ^III-Rb-^Ubi or pSK were co-bombarded with infectious clones of: B) the MSV-B strain isolate VW; C) the MSV-C strain isolate Set and D) the PanSV-A strain isolate Kar. The number of replicates for B) were: pSK, 12; pSPLIT*rep* ^III-Rb-^Ubi, 15. The number of replicates for C) were: pSK, 17; pSPLIT*rep* ^III-Rb-^Ubi, 26. The number of replicates for D) were: pSK, 12; pSPLIT*rep* ^III-Rb-^Ubi, 17. All real-time PCRs were performed on total DNA extracted from BMS cells four days post-bombardment.

**Figure 7:**
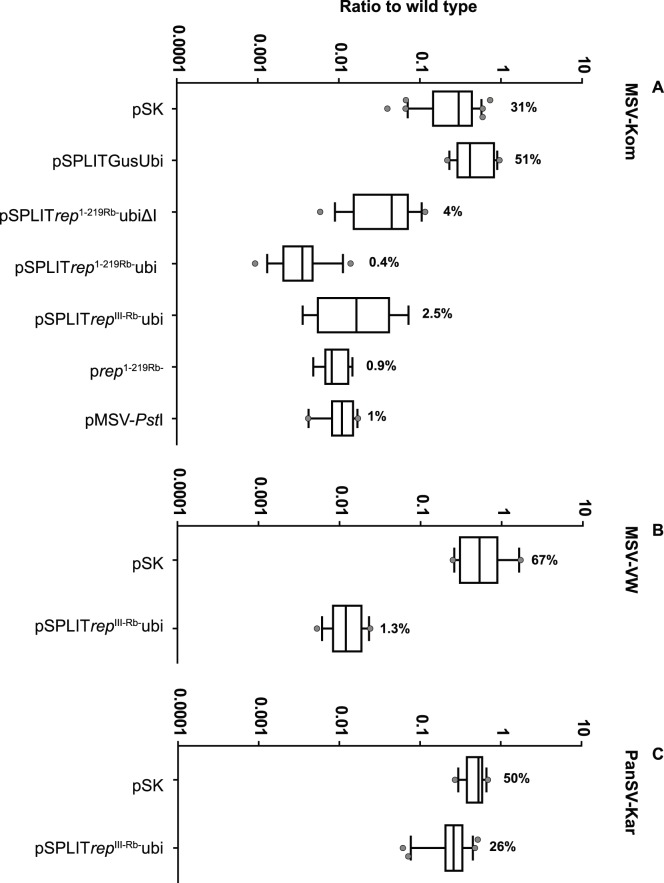
Vertical box-and-whisker plots summarising real-time PCR data on all constructs bombarded at a 5∶1 weight ratio with infectious clones of diverse MSV strains and another mastrevirus species. A) MSV-Kom. The plots were constructed as in Figure 6. The number of replicates (i.e. the number of bombarded samples) were as follows: pSK, 34; pSPLITGusUbi, 18; pSPLIT*rep* ^1-219Rb-^UbiΔI, 18; pSPLIT*rep* ^1-219Rb-^Ubi, 14; pSPLIT*rep* ^III-Rb-^Ubi, 8; p*rep* ^1-219Rb-^, 9; pMSV-*Pst*I, 11. Plots in B-C) were constructed as described for A), but this time either pSPLIT*rep* ^III-Rb-^Ubi or pSK were co-bombarded with infectious clones of: B) the MSV-B strain isolate VW; and C) the PanSV strain A isolate Kar. The number of replicates for B) were: pSK, 11; pSPLIT*rep* ^III-Rb-^Ubi, 14. The number of replicates for C) were: pSK, 16; pSPLIT*rep* ^III-Rb-^Ubi, 25. All real-time PCRs were performed on total DNA extracted from BMS cells four days post-bombardment.

Semi-quantitative end-point PCR demonstrated that all but the three GUS-based constructs inhibited viral replication to some extent (data not shown). Of all the promoter combinations, the ubiquitin promoter + ubi-1 exon/intron (Ubi) resulted in the best inhibition. Subsequent quantitative realtime analyses were therefore done on the Ubi- and UbiΔI based constructs only (Figs. 6 and 7).

To control for the possible inhibitory effects on viral replication of both the vector sequences surrounding the SGCs, and the SGC “backbone” itself, pSK and pSPLITGUSUbi were tested as negative controls alongside *rep*-containing SGCs. In addition, p*rep* ^1-219Rb-^ was used as a positive control against which the effectiveness of the SGCs could be compared.

Surprisingly, co-bombardment of pKom602 at a 1∶1 weight ratio with pSK resulted in a reduction in viral DNA levels compared with bombardment of pKom602 alone (wild type [wt] replication; see Fig. 6A). Average levels of pKom602 co-bombarded with pSK were reduced by 32% when compared with wt (P<0.0001; Wilcoxan signed rank test). Although viral levels were also reduced upon co-bombardment at a 1∶1 ratio with pSPLITGUSUbi, the difference from wt was minor (14% reduction; Fig. 6A) and not significant (P = 0.2783 Wilcoxan signed rank test). Importantly, the difference between the GUS and pSK datasets was also not significant (P = 0.0936; Mann Whitney test). Levels of viral DNA after co-bombardment with pSK were therefore taken as the baseline, and any significant reductions beyond these levels were interpreted as being due to inhibition by the *rep*-containing SGCs (unless otherwise stated, given P values, calculated using a Mann Whitney test followed by a step down multiple testing correction, are from comparisons between [virus + SGC] and [virus + pSK] datasets).

Since p*rep* ^1-219Rb-^ had proven to be effective in transgenic plants when constitutively expressed [17], pSPLIT*rep* ^1-219Rb-^Ubi and pSPLIT*rep* ^1-219Rb-^UbiΔI, were the first to be assayed by qPCR (Fig. 6A). Viral inhibition was less effective from the SGCs than from the constitutively expressed construct. pSPLIT*rep* ^1-219Rb-^Ubi and pSPLIT*rep* ^1-219Rb-^UbiΔI resulted in a 75% reduction (P<0.0011) and a 51% reduction (P = 0.027) respectively in MSV-Kom levels, compared with 99% inhibition (P<0.0011) achieved by p*rep* ^1-219Rb-^. This is probably due to the fact that expression from the SGCs first has to be induced by the virus before the protein can inhibit viral replication.

Having determined that the Ubi-containing SGC resulted in the best virus-induced replication inhibition by *rep* ^1-219Rb-^ (indicating that the presence of the ubi-1 exon/intron did not interfere with splicing of the SGC syntron), we subsequently further tested only Ubi-containing constructs, starting with pSPLIT*rep* ^III-Rb-^Ubi. Previously, constitutively expressed Rep^III-Rb-^, a full-length Rep with mutations in RCR motif III and the pRBR interaction domain, completely inhibited viral replication in cell culture and resulted in immune *D. Sanguinalis* transgenic plants in challenge experiments [17]. However, constitutive expression induced growth and developmental defects in transgenic plants, possibly due to non-mutated motifs in the full-length Rep^III-Rb-^ protein (see Fig. 1) interacting with host regulatory molecules. This gene was therefore an ideal candidate for the virus-induced split gene system.

pSPLIT*rep* ^III-Rb-^Ubi inhibited MSV-Kom replication by 94% (P<0.0011) (Fig. 6A), a significant improvement over the truncated *rep* SGC (P<0.0011 between pSPLIT*rep* ^III-Rb-^Ubi and pSPLIT*rep* ^1-219Rb-^Ubi datasets) and much closer to the replication inhibition (99%) seen by the constitutively expressed p*rep* ^1-219Rb-^ that was used to make our MSV-resistant transgenic maize lines. The difference in replication inhibition by p*rep* ^1-219Rb-^ and pSPLIT*rep* ^III-Rb-^Ubi was not significant (P = 0.0948).

Inhibition by p*rep* ^1-219Rb-^ was possibly complete; with the DNA levels detected most likely being due to residual unreplicated plasmid DNA that remains detectable by qPCR four days after bombardment [43], [46]. To account for this input DNA, we bombarded maize suspension cells with a cloned replication-deficient MSV mutant, pMSV-*Pst*I (described by Owor et al., [43]) at the same concentration as pKom602. Four days post bombardment, MSV-*Pst*I was detected at levels that were 100-fold lower than MSV-Kom – this was due to the fact that MSV-Kom could be replicationally released [24] from the plasmid in which it was cloned by its wt Rep protein, while a non-functional Rep expressed by MSV-*Pst*I prevented its release from the inoculated plasmid. MSV-Kom DNA levels detectable after co-bombardment with pSPLIT*rep* ^III-Rb-^Ubi were only marginally different (P = 0.055) from MSV-*Pst*I levels, and there was no difference at all from MSV-*Pst*I levels when MSV-Kom was co-bombarded with p*rep* ^1-219Rb-^ (both were present at 1% of wt; P = 0.8749).

### Viral replication inhibition correlates with split gene cassette dosage

To determine if the replication inhibition observed with the SGCs was a “dominant negative mutant” [47] based mechanism, the Ubi and UbiΔI-containing SGCs were bombarded at 5∶1 ratios to pKom602 to achieve over-expression of the mutant Reps relative to viral gene expression (in effect simulating a low-pressure viral infection) (see Fig. 7).

As with a 1∶1 bombardment ratio, there was once again a marked decrease in MSV-Kom levels when co-bombarded with five-fold more pSK (Fig. 7A), this time by 69% (a 2.4-fold significant decrease relative to when they were co-bombarded at a 1∶1 ratio; P<0.0001). Similarly, five-fold more pSPLITGUSUbi decreased MSV-Kom DNA levels by 49% (a statistically significant 3.5-fold decrease relative to when they were co-bombarded at a 1∶1 ratio; P = 0.0075).

Since pSPLITGUSUbi contains two copies of the MSV-Kom LIR and should be replicationally released from the pSK backbone by MSV-Kom Rep, the decrease in viral replication could be due to competition for the viral Rep at Rep binding sites in both the SGC and MSV-Kom LIRs – the former outnumbering the latter. However, this does not explain the greater inhibition of replication seen by pSK at both 1∶1 and 5∶1 ratios.

To account for this “non-specific” viral inhibition, decreases in viral DNA levels when co-bombarded with *rep*-containing SGCs were compared with MSV-Kom + pSK levels, and only significant differences were taken as being due to the mutant Reps.

Even taking into account the increased inhibition by pSK, viral replication was inhibited significantly more when the SGCs were bombarded at a 5∶1 ratio with pKom602 compared with at a 1∶1 ratio (Fig. 7A). Compared with a 75% reduction at a 1∶1 SGC:virus ratio, viral levels were reduced by 99.6% in the presence of five-fold more pSPLIT*rep* ^1-219Rb-^Ubi (P<0.0001). Similarly, reduction in DNA levels by pSPLIT*rep* ^1-219Rb-^UbiΔI went from 51% (1∶1 ratio) to 96% (5∶1 ratio) (p<0.0001). Compared with a 1∶1 SGC:virus ratio, the difference between the ubiΔI- and ubi-containing SGCs was more marked at a 5∶1 ratio (25-fold vs 236 fold inhibition by pSPLIT*rep* ^1-219Rb-^UbiΔI and pSPLIT*rep* ^1-219Rb-^Ubi respectively; P<0.0001). This was expected given that the presence of the ubi-1 intron is known to enhance expression from the ubi promoter (intron mediated enhancement [48]).

The effect of bombarding five-fold more transgene DNA was not as great with p*rep* ^1-219Rb-^ (99% reduction in viral DNA levels at both 1∶1 and a 5∶1 ratios; P = 0.4363) or with pSPLIT*rep* ^III-Rb-^Ubi (94% reduction at a 1∶1 ratio and 97.5% reduction at a 5∶1 ratio; P = 0.0603). This is understandable for p*rep* ^1-219Rb-^ considering that at a 1∶1 ratio, detected amplicons were probably from input plasmid and not replicated viral DNA, hence there would be no difference in input pKom602 DNA levels whether bombarded at a 5∶1 or 1∶1 ratio. For pSPLIT*rep* ^III-Rb-^Ubi, the already low levels of viral DNA detected at a 1∶1 ratio were reduced so as to make them indistinguishable from input DNA levels.

### The effectiveness of pSPLIT*rep* ^III-Rb-^Ubi against diverse viral strains or species is associated with their degree of sequence similarity to MSV-Kom

The mutant *rep* transgenes used in the SGCs were derived from the isolate, MSV-Kom, which belongs to the A-strain of MSV that causes the most severe form of maize streak disease [28]. MSV-Kom, first isolated from maize in Komatieport, South Africa [40] belongs to an MSV-A subtype known as MSV-A_4_, which is the most prevalent subtype found in South Africa [41] Having determined that the most effective SGC in inhibiting replication of MSV-Kom was pSPLIT*rep* ^III-Rb-^Ubi (Figs. 6A and 7A), we subsequently decided to test pSPLIT*rep* ^III-Rb-^Ubi against MSV isolates belonging to the B and C strains of MSV: (1) MSV-VW, belonging to the MSV-B strain (originally isolated from wheat but representative of viruses normally found infecting *Digitaria* sp. [49]); and (2) MSV-Set, belonging to the MSV-C strain (isolated from a *Setaria* plant and apparently representative of other *Setaria*-adapted MSV isolates [40]). MSV-VW and MSV-Set respectively share 89% and 78% genome-wide nucleotide similarity with MSV-Kom, and 86.5% and 81.4% Rep (with gaps included as a 21st character state) amino acid identity with MSV-Kom Rep. We also tested the construct against a different African streak virus species, PanSV-Kar, which shares 60% genome-wide nucleotide similarity with MSV-Kom and 60.6% Rep amino acid identity with MSV-Kom Rep.

Although MSV-VW, MSV-Set and PanSV-Kar are wild-grass-adapted virus isolates that do not cause serious disease in maize, we deemed it important to test pSPLIT*rep* ^III-Rb-^Ubi against these diverse isolates because efficient trans-replication by MSV Rep requires the presence of specific Rep-binding sites (replication specificity determinants, or RSDs) within the LIR [30]. Considering that expression of the transgene from the SGC requires the replicational release of the cassette by the viral Rep which is initiated by the binding of the Rep to these specific sites in the LIR, we sought to determine if this would occur in isolates with non-conserved RSDs. According to Willment et al. [30], while PanSV-Kar is more genetically divergent from MSV-Kom than MSV-Kom is to MSV-Set, the Rep from PanSV-Kar complemented the replication function of a Rep-deficient MSV-Kom genome more efficiently than did MSV-Set’s Rep. Efficient trans-replication presumably requires the sharing of RSDs within the LIR: Willment et al. [30] found that the RSDs in the MSV-Kom LIR are indeed more like PanSV-Kar’s than MSV-Set’s in terms of both spacing and sequence.

As can be seen in Fig. 6B–D, replication inhibition of all three divergent viruses did occur, but to a much lesser extent than inhibition of MSV-Kom. When co-bombarded at a 1∶1 SGC:virus ratio, pSPLIT*rep* ^III-Rb-^Ubi inhibited MSV-VW by 66%, MSV-Set by 73% and PanSV-Kar by 48%. However, unlike with MSV-Kom and MSV-Set, co-bombardment of pSK did not lead to a reduction in MSV-VW levels (Fig. 6B). Levels of MSV-Kom and MSV-Set DNA were 68% and 64% of wt in the presence of pSK, while levels of VW were 90%. Thus the reduction in MSV-VW DNA levels achieved by pSPLIT*rep* ^III-Rb-^Ubi when compared with wt (MSV-VW bombarded alone) should be put into context with viral DNA levels in the presence of pSK. Compared with virus + pSK levels, pSPLIT*rep* ^III-Rb-^Ubi reduced MSV-Kom titres by 91% (P<0.0001); MSV-VW by 63% (P<0.0001); MSV-Set by 57% (P<0.0001) and PanSV-Kar by 41% (P = 0.0178). This correlates well with the percent identity of each virus’ Rep with MSV-Kom. (MSV-Kom>MSV-VW>MSV-Set>PanSV-Kar).

Interestingly, these results do not correlate with the trans-replication efficiencies of MSV-Kom by MSV-Set and PanSV Reps described by Willment et al. [30]. It must be borne in mind that trans-replication of the MSV-Kom-derived SGC by these divergent viruses is only the first step in the viral replication inhibition process. Once the exon 1 and exon 2 of the *rep* ^III-Rb-^ are spliced together and Rep^III-Rb-^ is expressed, presumably there needs to be enough sequence identity between the Kom-derived mutant Rep and the inoculated virus’ Reps for trans-dominant negative mutant inhibition to occur. Evidence from challenges of our transgenic lines in which the *rep* ^1-219Rb-^ transgene was silenced or expression was reduced indicates that resistance is dependent on the expression level of the transgene (unpublished data). Considering the greater replication inhibition achieved by pSPLIT*rep* ^III-Rb-^Ubi when bombarded at a 5∶1 rather than a 1∶1 ratio with MSV-Kom, it is likely the same would apply for this transgene, and that the resistance mechanism is dependent on the mutant Rep “flooding” the inoculated virus-derived Rep, perhaps forming dysfunctional oligomers with the wt Rep and/or RepA [50]–[53]) or outcompeting wt Rep for iterated binding sites on the MSV genome.

To test this with the heterologous viruses, MSV-VW (which has the highest identity with MSV-Kom) and PanSV-Kar (which has the lowest identity), each virus was co-bombarded with pSPLIT*rep* ^III-Rb-^Ubi at a 5∶1 SGC:virus ratio. The replication of both viruses was drastically reduced compared with that observed at a 1∶1 ratio: MSV-VW replicated to only 1.3% of wt (P<0.0001), and PanSV to 26% of wt (P<0.0001), representing a 75-fold and 4-fold inhibition respectively (Fig. 7B and C).

## Conclusion

We have shown for the first time that the INPACT inducible hyper-expression platform, developed primarily for farming recombinant proteins in plants, can be adapted for virus-inducible resistance to MSV, with potential application in transgenic maize. While replication inhibition was greater with increased dosage of all the SGCs, one construct (pSPLIT*rep* ^III-Rb-^Ubi) was extremely effective even at the lower of the two doses that were tested, inhibiting replication of MSV-Kom by 94% when bombarded at a 1∶1 ratio with the virus. This construct was also effective against diverse MSV strains and even a different mastrevirus species, although the degree of replication inhibition correlated with the degree of sequence similarity to MSV-Kom (the isolate on which the SGCs were based).

The Rep-inducible nature of the INPACT platform has been demonstrated in transgenic tobacco for the high-level, activatable expression of four different recombinant proteins, including the lethal ribonuclease, barnase [18]. In contrast, it is difficult to definitively prove this using the micro-projectile bombardment transformation system and the SGCs utilised in this study. Firstly, in the absence of MSV Rep protein, *in situ* recombination of the SGC (most likely at the repeated stem-loop sequences) may occur in a small number of cells [20], [54]–[56] and this, in turn, could generate an episomal form of the cassette from which Rep can be expressed. Despite this possibility we were unable to detect SGC-encoded mutant *rep* transcripts in the absence of the MSV Rep protein (data not shown), suggesting these transcript levels are below the limit of detection by qPCR. Secondly, in the presence of MSV Rep protein it is anticipated that the expressed SGC-encoded mutant *rep* gene product inhibits MSV replication, thereby reducing both Rep forms in the system. Similarly, we were unable to detect SGC-encoded mutant *rep* transcripts under these circumstances (data not shown). Ultimately, the practicality of the SGCs described in this study will only be fully realised with the regeneration of phenotypically normal transgenic maize plants engineered to contain the SGC that are resistant/immune to MSV infection. To this end we have regenerated a number of transgenic maize lines containing a SGC capable of expressing the most effective Rep mutant, namely Rep^III-Rb-^. In contrast to lines constitutively expressing this mutant gene, SGC lines have produced T_2_ generation offspring with normal phenotypes.

Considering that only one strain - MSV-A - causes severe disease in maize throughout the whole geographical range of MSV, and that all isolates so far discovered within this strain have a maximum divergence of only 4.62% at the nucleotide level, it is likely that this novel MSV-inducible resistance construct will be effective against the complete spectrum of severe maize streak disease-causing African MSVs.

## Supporting Information

Figure S1 **Annotated sequence of the synthesised pSPLIT** ***rep*** **^1-219Rb-^35S, including important RE sites used for subsequent cloning.**
(DOC)
