# Supplementary material for: Inducible Resistance to Maize Streak Virus
Source: PLoS One. 2014 Aug 28;9(8):e105932. doi: 10.1371/journal.pone.0105932 (PMC4148390; doi:10.1371/journal.pone.0105932)
Supplement: Figure S1 — Annotated sequence of the synthesised pSPLIT rep 1-219Rb-35S, including important RE sites used for subsequent cloning. (DOC) [file pone.0105932.s001.doc]

**Supplementary Figure 1**

pSPLIT*rep*^1-219Rb-^35S (Synthesised). 2236 bp (excluding vector backbone).

**NotI** MSV-Kom LIR241

1 **GCGGCCGC**agccgacgacggaggttgaggctgagggatggcagactgggagctccaaact

61 ctatagtatacccgtgcgccttcgaaatccgccgctccattgtcttatagtggttgtaaa

121 tgggccggaccgggccggcccagcaggaaaagaaggcgcgcactaatattaccgcgcctt

181 cttttcctgcgagggcccggtagggaccgagcgctttgatttaaagcctggttctgcttt

**SwaI** Syntron 3’ half Rep^1-219Rb-^ exon2

241 gtatg**atttaaat**ctaactttttattttttattttgttttcttgc**agGC**ACATAAAGATG

301 GAAGTTTGCATTTACATGCATTGCTTCAGACAGAGAAGCCGATAAGGATATCTGACTCAA

361 GGTTCTTTGATATAAATGGGTTTCACCCAAATATTCAGAGTGCCAAGTCAGTTAACAGAG

421 TGAGGGATTACATTCTCAAGGAACCTCTGGCTGTGTTTGAGAGAGGTACTTTCATTCCTA

481 GGAAGTCCCCCTTCCTAGGAAAATCTGATTCAGAGGTAAAGGAAAAAAAGCCTTCTAAAG

541 ATGAAATAATGCGAGACATTATTTCACACGCTACCTCCAAAGAAGAGTACCTCTCCATGA

601 TCCAGAAAGAGCTCCCCTTTGATTGGTCCACAAAATTGCAGTATTTTGAATACTCTGCAA

661 ATAAGCTTTTTCCTGAGATTCAGGAAGAGTTCACCAATCCTCATCCACCCTCCTCACCTG

721 ATTTACTTTGTCTTAAGTCAATCAACGATTGGCTCCAGCCTAACATCTTCCAGGTTAGTC

pSK seq 3’ of rep **SpeI** Nos Terminator

781 CCGAAGCT**TGA**TATCGAATTCCTGCAGCCCGGG**ACTAGT**CGTTCAAACATTTGGCAATAA

841 AGTTTCTTAAGATTGAATCCTGTTGCCGGTCTTGCGATGATTATCATATAATTTCTGTTG

901 AATTACGTTAAGCATGTAATAATTAACATGTAATGCATGACGTTATTTATGAGATGGGTT

961 TTTATGATTAGAGTCCCGCAATTATACATTTAATACGCGATAGAAAACAAAATATAGCGC

XhoI

1021 GCAAACTAGGATAAATTATCGCGCGCGGTGTCATCTATGTTACTAGATCGGGCTCGAGTG

MSV-Kom SIR

1081 AATAAAAACTCCCGTTTTATTATATTTGATGAATGCTGAAAGCTTACATTAATATGTCGT

1141 GCGATGGCACGAAAAAACACACGCAAACAATACAGGGGGGTAGTCGGCGGGCGGCTAAGG

**AscI**

1201 GTGGTGCTCGGCGGGCAGAACATCGAAAAATCAAGATCTATATGAA**GGCGCGCC**TCATGG

CaMV35 S Promoter

1261 AGTCAAAGATTCAAATAGAGGACCTAACAGAACTCGCCGTAAAGACTGGCGAACAGTTCA

1321 TACAGAGTCTCTTACGACTCAATGACAAGAAGAAAATCTTCGTCAACATGGTGGAGCACG

1381 ACACACTTGTCTACTCCAAAAATATCAAAGATACAGTCTCAGAAGACCAAAGGGCAATTG

1441 AGACTTTTCAACAAAGGGTAATATCCGGAAACCTCCTCGGATTCCATTGCCCAGCTATCT

1501 GTCACTTTATTGTGAAGATAGTGGAAAAGGAAGGTGGCTCCTACAAATGCCATCATTGCG

1561 ATAAAGGAAAGGCCATCGTTGAAGATGCCTCTGCCGACAGTGGTCCCAAAGATGGACCCC

1621 CACCCACGAGGAGCATCGTGGAAAAAGAAGACGTTCCAACCACGTCTTCAAAGCAAGTGG

1681 ATTGATGTGATATCTCCACTGACGTAAGGGATGACGCACAATCCCACTATCCTTCGCAAG

**BamHI**

1741 ACCCTTCCTCTATATAAGGAAGTTCATTTCATTTGGAGAGAACACGGGGGACTCTTGA**GG**

Rep^1-219Rb-^ Exon 1

1801 **ATCC*ATG***GCCTCCTCCTCATCCAACCGTCAGTTCTCACACAGGAACGCTAACACGTTCCT

1861 AACCTATCCAAAGTGTCCAGAAAATCCTGAAATCGCCTGTCAGATGATCTGGGAGCTCGT

Syntron 5’ half

1921 CGTTCGTTGGATTCCCAAATATATTCTATGTGCCCGAG**AGgt**aagatttttattttttat

**PacI** MSV-Kom LIR241

1981 **ttaattaa**agccgacgacggaggttgaggctgagggatggcagactgggagctccaaact

2041 ctatagtatacccgtgcgccttcgaaatccgccgctccattgtcttatagtggttgtaaa

2101 tgggccggaccgggccggcccagcaggaaaagaaggcgcgcactaatattaccgcgcctt

2161 cttttcctgcgagggcccggtagggaccgagcgctttgatttaaagcctggttctgcttt

**KpnI**

2221 gtatgattta**GGTACC**
